# Evidence in Sheep for Pre-Natal Transmission of Scrapie to Lambs from Infected Mothers

**DOI:** 10.1371/journal.pone.0079433

**Published:** 2013-11-18

**Authors:** James D. Foster, Wilfred Goldmann, Nora Hunter

**Affiliations:** The Roslin Institute and Royal (Dick) School of Veterinary Studies, The University of Edinburgh, Easter Bush, Midlothian, Scotland, United Kingdom; University of Maryland School of Medicine, United States of America

## Abstract

Natural scrapie transmission from infected ewes to their lambs is thought to occur by the oral route around the time of birth. However the hypothesis that scrapie transmission can also occur before birth (*in utero*) is not currently favoured by most researchers. As scrapie is an opportunistic infection with multiple infection routes likely to be functional in sheep, definitive evidence for or against transmission from ewe to her developing fetus has been difficult to achieve. In addition the very early literature on maternal transmission of scrapie in sheep was compromised by lack of knowledge of the role of the *PRNP* (prion protein) gene in control of susceptibility to scrapie. In this study we experimentally infected pregnant ewes of known *PRNP* genotype with a distinctive scrapie strain (SSBP/1) and looked for evidence of transmission of SSBP/1 to the offspring. The sheep were from the NPU Cheviot flock, which has endemic natural scrapie from which SSBP/1 can be differentiated on the basis of histology, genetics of disease incidence and strain typing bioassay in mice. We used embryo transfer techniques to allow sheep fetuses of scrapie-susceptible *PRNP* genotypes to develop in a range of scrapie-resistant and susceptible recipient mothers and challenged the recipients with SSBP/1. Scrapie clinical disease, caused by both natural scrapie and SSBP/1, occurred in the progeny but evidence (including mouse strain typing) of SSBP/1 infection was found only in lambs born to fully susceptible recipient mothers. Progeny were not protected from transmission of natural scrapie or SSBP/1 by washing of embryos to International Embryo Transfer Society standards or by caesarean derivation and complete separation from their birth mothers. Our results strongly suggest that pre-natal (*in utero*) transmission of scrapie may have occurred in these sheep.

## Introduction

Scrapie is one of the transmissible spongiform encephalopathies (TSE) or prion diseases. It occurs naturally in sheep and goats but can also be transmitted by experimental inoculation. TSEs are found in other animal species including Chronic Wasting Disease (CWD) in deer and Bovine Spongiform Encephalopathy (BSE) in cattle. There are also human forms of TSEs such as Creutzfeldt- Jacob Disease (CJD), Gerstmann-Straussler Syndrome (GSS) and variant CJD (vCJD), the latter being the result of inadvertent infection of humans with BSE. Although characteristics of any particular TSE can differ, there are broad similarities amongst them and results from studies of disease susceptibility and progression in one species may apply also to others.

TSEs exist in a range of different strains, characterised by the formation of a mis-folded isoform of the prion protein (PrP^Sc^) which is relatively proteinase resistant compared with the normal protein, PrP^C^. Susceptibility to TSEs in sheep is controlled by alleles of the gene encoding PrP, known as *PRNP*, and although much is now understood about the pathogenesis and strain targeting of different *PRNP* genotypes, the transmission of natural scrapie from sheep to sheep is not fully understood. Early researchers noticed that scrapie often occurred in sheep which were the offspring of scrapie affected mothers [1] and the idea of *in utero* maternal transmission of infection was suggested, however this is difficult to differentiate from transmission via close contact after birth in the perinatal period. More recently the interaction between sheep *PRNP* genotype and incidence of natural and experimental scrapie was elucidated [2], [3] and as scrapie is not a genetic disease [4], [5] the perception of maternal transmission is now realised to be at least partly the result of inheritance of *PRNP* alleles specifying susceptibility to a prevalent infection. It is also clear that any experimental studies of clinical scrapie in ewes and their lambs must involve animals of known *PRNP* genotype to be truly informative and therefore many of the very early studies, carried out before the PrP protein was discovered, are now of limited usefulness. However two of these, one in Scotland, UK [1] and the other in the US [6], supported the idea of mother to offspring transmission of scrapie in sheep.

*PRNP* genetics is complex in sheep however codons 136, 154 and 171 are of major importance and genotypes range from the highly susceptible VRQ/VRQ through to the less susceptible ARQ/ARQ and on to the highly resistant ARR/ARR, although the details are dependent on the infecting strain of scrapie. Recent scrapie studies in sheep using such genetic analysis are tantalizing in possibly showing low levels of *in utero* transmission but lacking in clear evidence [7], [8], [9], [10] and currently the most prevalent view is that contact transmission around the time of birth between infected ewe and her lamb is the most likely route of infection.

The sheep placenta, after voiding, has been implicated in the transmission of disease via contact with lambs, because it is known to contain infectivity [11], [12] detectable by mouse bioassay. A quicker method of investigating which tissues may carry infectivity is by detection of the scrapie specific isoform of the prion protein, PrP^Sc^, and this has also been found in placental samples [11], [13]. It has been noted that PrP^Sc^ accumulates in placentas where the conceptus is of susceptible genotype and not when it is resistant [14], [15], except in the specific instance where a resistant fetus develops in the same uterine horn as a susceptible sibling [16]. This process is also dependent on the *PRNP* genotype of the ewe (eg VRQ/VRQ, ARQ/ARQ) as scrapie infected VRQ/ARR animals were not found to have PrP^Sc^ in placenta even when the fetus was susceptible [17]. However as no PrP^Sc^ was found in susceptible fetuses in late gestation even when the placenta was PrP^Sc^ positive, it was concluded [14] that it was most likely that transmission of infection was occurring, not *in utero* but via transfer of infection from voided placentae to the lambs immediately after birth. Other sources of infection have been implicated including amniotic fluids [18] and milk [19], [20], [21] and as very young lambs are highly susceptible to prion infection [22] it is clear that the peri-natal period after birth is one of high risk of scrapie transmission.

There remains considerable interest in whether or not the sheep embryo and/or fetus become infected via the germ line or from its mother *in utero* and whether it can subsequently carry infection to its surrogate mother. The International Embryo Transfer Society (IETS) reports a small but steady collection and transfer of sheep embryos for breeding purposes [23]. In 2010 the global statistics reported that 32,614 viable sheep embryos were flushed and transferred, mostly in Australia, followed by the Republic of South Africa and South America and very low numbers in Europe and North America. Most of the embryos are used fresh but there are reported instances of international trade in frozen embryos and all of these procedures could facilitate spread of scrapie if embryos were shown to be at risk. In recent years with the development of highly sensitive detection methods such as Protein Misfolding Cyclic Amplification (PMCA), evidence of low amounts of PrP^Sc^ has been found in in ram semen [24] and also in amniotic fluid and susceptible fetuses [18]. However low levels of infectivity do not always result in disease and it is important also to demonstrate that clinical signs of scrapie do, or do not, result. In an experimental study using six day old embryos taken from ewes exposed to natural scrapie and then transferred to resistant recipients, we were able to produce a clean uninfected flock which did not develop scrapie [25] a result which suggests that embryos may be clean of infection. Therefore if infection does transfer to offspring before birth, it must do so at later stages of gestation. Exactly when this transmission may occur is debatable however there is one report of scrapie occurring in a lamb which was born to a scrapie-affected mother by caesarean derivation. The lamb had no contact with the mother after birth and was kept in isolation and fed milk replacers, however it did itself die of scrapie aged 6 months. It is difficult in this case to see where the infection came from, unless via the mother *in utero* [26]. One single uncontrolled observation is not convincing evidence however a recent report of CWD studies in Muntjac deer found both disease-related PrP in foetal tissues and CWD clinical disease appearing in other offspring of infected mothers [27]. The authors suggest that this is evidence for *in utero* transmission of this TSE and that this route of infection may be underestimated for all TSEs.

To add to the body of evidence in support of pre-natal TSE transmission, we report here a study of experimental and natural scrapie infected sheep, which was started in 1994 and took over 15 years to complete. The sheep were part of the Neuropathogenesis Unit (NPU) Cheviot flock in which only animals of *PRNP* genotypes VRQ/VRQ and VRQ/ARQ succumb to natural scrapie (NPU-scrapie) which is clinically and pathologically distinct from scrapie experimentally induced by inoculation with the SSBP/1-scrapie source [28], [29], [30], [31], [32]. The aim was to study transmission of experimental SSBP/1 scrapie between ewes and lambs using embryo transfer to allow us to place lambs in recipients of a wide range of *PRNP* genotype and therefore disease susceptibility. A proportion of the embryos was washed according to IETS recommendations and a proportion of recipients was challenged with SSBP/1 scrapie. We then used caesarean derivation to remove some lambs from peri-natal contact with their surrogate mothers and therefore to reduce any horizontal spread of infection from ewe to lamb. Cases of scrapie in the offspring, particularly the caesarean derived animals, strongly suggest that pre-natal transmission of scrapie infection can occur in sheep.

## Methods

### Ethics Statement

The experiments in sheep were approved by the Institute for Animal Health Protocols and Ethics Committee in 1994 and carried out under licence from the UK Home Office (30/4301 and 60/4613). All surgery was performed under appropriate anaesthesia by experienced and licenced personnel. Animals developing clinical signs were humanely culled once signs were confirmed and according to UK Home Office approved procedures. Animals developing intercurrent illness were treated by veterinary surgeons and culled humanely as above if there was no response to treatment. The experiments using tg338 mice were carried out in Toulouse, France in accordance with the European Community Council Directive 86/609/EEC and were approved by the INRA Toulouse/ENVT committee.

### Sheep and *PRNP* Genotypes

Sheep were South Country Cheviots from the NPU flock (NPU Cheviots) which has been bred since 1960 into two genetically discrete lines: the positive line being susceptible to experimental subcutaneous challenge with SSBP/1 scrapie, and the negative line which resists such challenge [2]. The two lines of sheep differ in frequencies of *PRNP* genotypes which are described for codons 136, 154 and 171 and for each allele in turn using the single letter code for each amino acid as follows: A – alanine, V – valine (codon 136); R – arginine, H – histidine (codon 154); R – arginine, Q – glutamine (codon 171). In the early stages of this study (∼1994) some of the embryo donor sheep had only had codon 136 established and because of lack of material it is not now possible to obtain full genotype information. Therefore in some cases, X is used to represent either of the two possibilities at codons 154 and 171. The positive line carries a high frequency of the allele VRQ_,_ while the negative line is a mixture of AXX/AXX genotypes. Natural scrapie occurs in the positive line in the genotypes VRQ/VRQ and VRQ/ARQ_,_ and has never appeared in the negative line.

*PRNP* genotyping methods for sheep have been described in detail previously. In a few cases sheep codons 136 and 154 were identified following *Bsp*H1 digestion of PCR-generated DNA fragments [2] and codon 171 by allele specific hybridisation [33]. However most genotypes were generated by automated DNA sequencing on an ABI Prism 377 DNA sequencer as described previously [34]. Some sheep were challenged subcutaneously with SSBP/1 scrapie as described previously [35] and conditions of care of the animals are given below.

To aid in the understanding of a very complex experiment, sheep have been given numerical identification numbers in the tables of results. With prefix D, sheep are donors (biological mothers) of embryos; with prefix R, sheep are recipients (surrogate mothers) of embryos; with prefix L, sheep are lambs, the offspring which developed from the transferred embryos.

### Embryo Transfer

All embryos were the result of mating by artificial insemination (AI) between 15 ewes and three rams (Table S1). Thirteen of the donor ewes encoded at least one VRQ allele as follows: D1 was VRQ/VRQ, four (D2–D5) were VRQ/ARQ, seven (D6–D12) were VRQ/AHQ, one (D13) was VRQ/ARR. D14 was ARQ/AHQ and D15 was ARQ/ARR. All were super ovulated with follicle stimulating hormone and synchronised in oestrus to the recipients with progestagen impregnated sponges. Three rams, one VRQ/ARQ (A) and two VRQ/ARR (B and C) were used for artificial intra-uterine insemination and embryos were transferred into recipients at 6 days of age in a process described previously [36]. The effect of embryo washing on subsequent development of scrapie in the resulting lambs was tested using the procedure of the International Embryo Transfer Society (IETS) [37]. A proportion of the embryos was transferred through five washes of phosphate-buffered saline (PBS), two washes containing 0.25% trypsin, followed by five final washes through PBS. Following a check for intact zona pellucida, washed and unwashed embryos were transferred into recipients by intra-uterine laparoscopy, a procedure carried out by specialist veterinarians from Edinburgh Genetics [36], a company which no longer exists, however a similar service is available from Innovis in the UK (www.innovis.org.uk).

### Scrapie Challenges of Sheep

One group of recipients (controls) was not inoculated with scrapie (Group 1). Recipient sheep in two other groups (Groups 2 and 3) were challenged subcutaneously (sc) with SSBP/1 (2 mls of 10^−1^ brain homogenate in saline) 26 days prior to the embryo transfer (ET). Group 2 recipients were of *PRNP* genotypes shown by us previously to be resistant to SSBP/1 whereas Group 3 recipients were of *PRNP* genotypes susceptible to SSBP/1 [38]. At post mortem, brain and spleen were collected and either fixed in formal saline for histology/immunohistochemistry or quickly frozen for Western blot analysis. In a few cases voided placental tissues were collected for immunohistochemistry.

### Lambing and Sheep Care

Lambing took place in 1994 and 1995 in pens cleaned prior to use with a power washer using hot water and disinfected with sodium hypochlorite solution (chloros) providing at least 20,000 ppm available chlorine. A number of recipient ewes in each group were enclosed individually in separate pens and lambed naturally. Placental tissue was removed where possible and, if clean, some samples were taken for analysis. The lambs were suckled by their mothers. The remaining recipients had their lambs delivered by caesarean section (midline laparotomy) by specialist veterinarians (Edinburgh Genetics) and these lambs had no further contact with the ewes but were given colostrum and then bottle-fed with milk powder, both derived from New Zealand and therefore judged to be free from TSE contamination.

All lambs were kept separated from all other sheep on the facility and maintained in groups according to whether their surrogate mothers had been challenged with SSBP/1 or not. Donors remained separate from the other sheep in the study after ET procedures and the recipients were also segregated in challenged and unchallenged groups. There was no nose-to-nose contact between sheep groups and all equipment necessary for husbandry procedures was dedicated to this study and kept separate for challenged and unchallenged groups. Staff caring for the animals followed strict disease hygiene procedures including walking in rubber boots through chloros foot baths before and after handling the animals. Boots and gloves were changed between challenged and unchallenged sheep. Caesarean-derived lambs from challenged recipients in pens separate from the other lambs and adults in the study and were hand reared by dedicated staff, different to those who cared for the lambs from unchallenged recipients and also following strict hygiene procedures. After weaning, all lambs were fed hay made from freshly grown pasture, which had had no sheep grazing on it since it was re-sown after several years of growing barley. When protein supplements were required, only vegetable based products were used. Where bedding was required this was either wood shavings or barley straw from arable farms with no contact from ruminants. The same disease hygiene procedures continued to be followed throughout the 15 years of the experiment.

### Histology and Western Blotting

Pathological examination of brain sections was performed routinely on all animals dying for any reason (scrapie or intercurrent illness). Immunocytochemistry (ICC) to detect PrP^Sc^ was performed using the streptavidin-biotin method with the mouse monoclonal antibody BG4: diluted 1∶100 [32], [39]. The secondary antibody was biotinylated rabbit anti-mouse IgG conjugated to streptavidin for staining with the chromagen AEC (aminoethyl carbazole) or DAB (diaminobenzidene). Scrapie positive control tissue came from previously confirmed natural scrapie sheep cases. For detection of the TSE related isoform of the PrP protein, PrP^Sc^, Western blotting using the polyclonal antibody 1B3 [40] was carried out as described [41].

### Distinction between Natural Scrapie and SSBP/1 Scrapie

The NPU Cheviot flock has endemic natural scrapie which is a potentially confounding factor however it is distinguishable from experimental SSBP/1 based on the features listed below.

1. *clinical signs*: NPU Cheviot natural scrapie has signs of ataxia and pruritus with a slow clinical course which can last up to four months, whereas in NPU Cheviots SSBP/1 scrapie has a faster onset and a short phase (a few days to 3–4 weeks) of ataxia with little sign of pruritus [32].
2. *vacuolation and levels of PrP^Sc^ immunostaining in the brain*: vacuolation is very sparse in SSBP/1-affected brain [42], much less severe than with natural scrapie [32], [43]. The lack of signs of SSBP/1 vacuolation pathology in affected sheep brain has been reported and commented upon by previous researchers and other labs since the 1950s [28], [29], [30], [31], [43]. Intensity of PrP^Sc^ immunostaining in SSBP/1 brain with the BG4 antibody is always marginal in the medulla following subcutaneous (sc) inoculation. The dorsal motor nucleus of the vagus (DMNV), always intensively stained in NPU-natural scrapie, is always more weakly stained and can sometimes be negative with SSBP/1. However, in thalamic nuclei, SSBP/1 generates a more widespread and intense staining whereas NPU-natural scrapie has very mild extra-cellular staining of the neuropil, although frequently with very intense peri-vascular deposits which are absent with SSBP/1 [32]. These differences between SSBP/1 and natural scrapie PrP^Sc^ pathology in sheep brain have also been reported by independent researchers using NPU Cheviot sheep [44] and other Cheviots with natural scrapie [45] and may be a feature of experimental challenges as the same phenomenon (low vacuolation and PrP^Sc^ levels) has been reported in the US in Suffolk sheep [46] challenged with scrapie and compared with naturally infected animals.
3. *PrP genotypes*: any sheep encoding at least one VRQ allele is susceptible to SSBP/1 [38] but only VRQ/VRQ and VRQ/ARQ are susceptible to NPU-natural scrapie [47]. Since 1981 when routine *PRNP* genotyping was started in the NPU flock there have to date been 245 cases of scrapie in VRQ/VRQ (around 90% of this genotype in the flock) and 78 in VRQ/ARQ (around 60% of this genotype in the flock) with none recorded in other VRQ-encoding genotypes which are maintained at similar levels in the population to VRQ/ARQ. Every sheep dying for any reason in the flock has been tested for PrP^Sc^ in brain and lymphoid tissue by immunohistochemistry from around 1992 onwards. In the main breeding NPU Cheviot flock the lambs born in the same 1994 and 1995 birth cohorts as the sheep involved in this study were kept completely separately from the study sheep (see above). There were in these 1994/1995 birth cohorts 8 VRQ/AHQ and 17 VRQ/ARR animals which lived to 4–7 (median 5) and 4–12 (median 6) years of age respectively with no sign of clinical scrapie, clinically or of PrP^Sc^ in brain or lymphoid tissues by immunohistochemistry. The occurrence of scrapie in VRQ/AHQ and VRQ/ARR sheep is therefore regarded as strong evidence for SSBP/1 infection rather than natural scrapie.
4. *transmission into tg 338 mice*: Tg338 mice [48] which express the ovine VRQ allele, have been used in several labs for transmission studies of natural scrapie. [49], [50], [51], [52], [53], [54]. They are particularly useful due to their high sensitivity to the so called “rapid strains” which produce very short incubation periods (∼60 days) following intracerebral challenge with a particular group of scrapie isolates [50], [51], [52] and much longer incubation periods with other groups of isolates [50], [51], [52], [55]. The rapid strains also produce distinctive pathology in the bioassay mouse brains [49]. From several independent transmissions in Roslin and INRA, SSBP/1 has been identified as a member of the rapid strain group producing very short incubation periods of between 50 and 60 days, whereas the natural scrapie from the NPU flock is one of the longer incubation period scrapie strains with incubation periods in excess of 370 days (Andreolletti and Hunter, this paper and unpublished).

SSBP/1 and NPU-natural scrapie are not easily distinguishable by Western blotting as their glycoform profiles are very similar, however protein analysis was carried out to help confirm the presence of the disease associated PrP^Sc^ protein.

Since the NPU flock was founded in 1960, there have been several hundred experimental transmissions of SSBP/1 but no evidence of natural spread of SSBP/1 or SSBP/1-like features of scrapie occurring in unchallenged animals [42], [47].

### Strain Typing by Mouse Bioassay

Samples from brain tissue of five scrapie affected offspring were strain typed in order to confirm the identity of the scrapie strain causing illness in the progeny. The bioassay was carried out in tg338 mice which express the sheep VRQ *PRNP* allele and are highly susceptible to SSBP/1 scrapie. Six mice were intra-cerebrally inoculated with each sample (20 µl). Prior to inoculation homogenate were diluted (final concentration 10%) in 5% glucose sterile solution. Each homogenate was tested for bacterial contamination (blood agar plates incubated overnight at 37C) and only sterile samples injected. The mice were injected and held in INRA facilities in Toulouse, France as at that time our laboratory in Edinburgh did not have tg338 mice. Mice were monitored daily until TSE clinical signs were noted and were then culled humanely. Formalin fixed brain was taken for vacuolar brain lesion profiling [56].

## Results

### Embryo Donors (Biological Parents)

Three female donors (D1, D2 and D5) developed natural scrapie after their embryos were collected (Table S1). In summary, D1 (VRQ/VRQ) developed natural scrapie aged 837 days, 174 days after embryo transfer (ET), whereas D2 and D5 (both VRQ/ARQ), developed natural scrapie aged 1216 days, 218 days after ET, and at age 1646 days, 1321 days after ET respectively. The 12 remaining embryo donors died with illnesses unrelated to scrapie between 1181 and 3977 days of age and 200–330 days after ET. The three rams all died of causes unrelated to scrapie at ages of around 2,000 days and over 1,000 days after being used for artificial insemination.

### The Effect of Embryo Washing

Some of the embryos were washed according to IETS recommendations [37] prior to transfer into recipient animals. There were cases of scrapie in sheep originating from both washed and unwashed embryos (summarised in Table S2). There are so many different categories within this study that group sizes are small, however the most useful information comes from fully susceptible offspring with biological mothers that were incubating natural scrapie. Of this group, three out of a total of seven (43%) washed as embryos, developed scrapie and six out of a total of ten (60%), not washed as embryos, developed scrapie, a non-significant difference (χ^2^ = 0.85, P = 0.27). In the washed group scrapie occurred in two offspring transferred into resistant recipients and one offspring transferred into a susceptible recipient. In the unwashed group all six scrapie affected offspring had been transferred into resistant recipients. No further details of this work are presented as embryo washing was judged not to have protected the resulting lambs from scrapie.

### Group 1: Unchallenged Recipient Sheep

Group 1 embryos were transferred into recipients of a range of *PRNP* genotypes and not challenged with SSBP/1. This control group was designed to indicate background levels of natural scrapie appearing in the offspring. Group1 recipients (n = 12) were of mixed PrP genotypes, eleven of them resistant to NPU-natural scrapie. Twelve recipients lived to ages between 1700 and 4324 days and died with no clinical, histological or immunohistochemistry (IHC) signs of scrapie. In contrast the thirteenth recipient, R16, showed mild PrP^Sc^ deposition in the dorsal motor nucleus of the vagus (DMNV), had genotype VRQ/ARQ but died with no scrapie clinical signs aged 2220 days. As VRQ/ARQ is a genotype which can be affected by NPU-natural scrapie, it was likely that sheep R16 was showing signs of sub-clinical scrapie (Table 1).

**Table 1:** Group 1: unchallenged recipients and development of scrapie in offspring.

Donor	Recipient	Genotype	Age died	Lamb born	Lamb	Genotype	Death scrapie	Death other
D5 (scr)*	R16	VRQ/ARQ	2220d	CD	L53	VRQ/ARQ		1474d
D5 (scr)	R17	VRQ/AHQ	3977d	CD	L54	VRQ/ARR**		3592d
D5 (scr)				CD	L55	VRQ/VRQ**	846d	
D11	R18	ARQ/ARR	2768d	CD	L56	AHQ/ARR		3592d
D11				CD	L57	VRQ/VRQ		2069d
D9	R19	AHQ/ARR	3204d	NB	L58	VRQ/AHQ		2969d
D4	R20	ARR/ARR	3075d	NB	L59	VRQ/ARR		4530d
D1 (scr)	R21	AXX/AXX	1700d	CD	L60	VRQ/ARQ		4515d
D1 (scr)				CD	L61	VRQ/ARQ	1863d	
D12	R22	AXX/AXX	3172d	NB	L62	VRQ/AHQ		4760d
D12	R24	AXX/AXX	1385d	NB	L64	VRQ/VRQ	954d	
D12				NB	L65	VRQ/AHQ		4907d
D15	R25	AXX/AXX	2567d	NB	L66	ARR/ARR		3812d
D15				NB	L67	ARR/ARR		4202d
D15	R26	AXX/AXX	1777d	NB	L68	VRQ/ARQ		4204d
D15				NB	L69	VRQ/ARQ		4204d
D15	R27	AXX/AXX	3052d	NB	L70	ARR/ARR		2575d
D15	R28	AXX/AXX	4324d	NB	L71	VRQ/ARQ		4268d
D15				NB	L72	ARR/ARR		4894d
Totals								
7	12			12NB, 7CD	19		3	16

CD = caesarean derivation and hand rearing, NB = natural birth, reared by recipient ewe.

* (scr) – embryo donor later developed natural scrapie

** Where two progeny indicated per recipient, these developed *in utero* together and born as “twins”. Deaths are from scrapie (Death scrapie) or other causes (Death other) and given in days of age (d).

**Figure 1:**
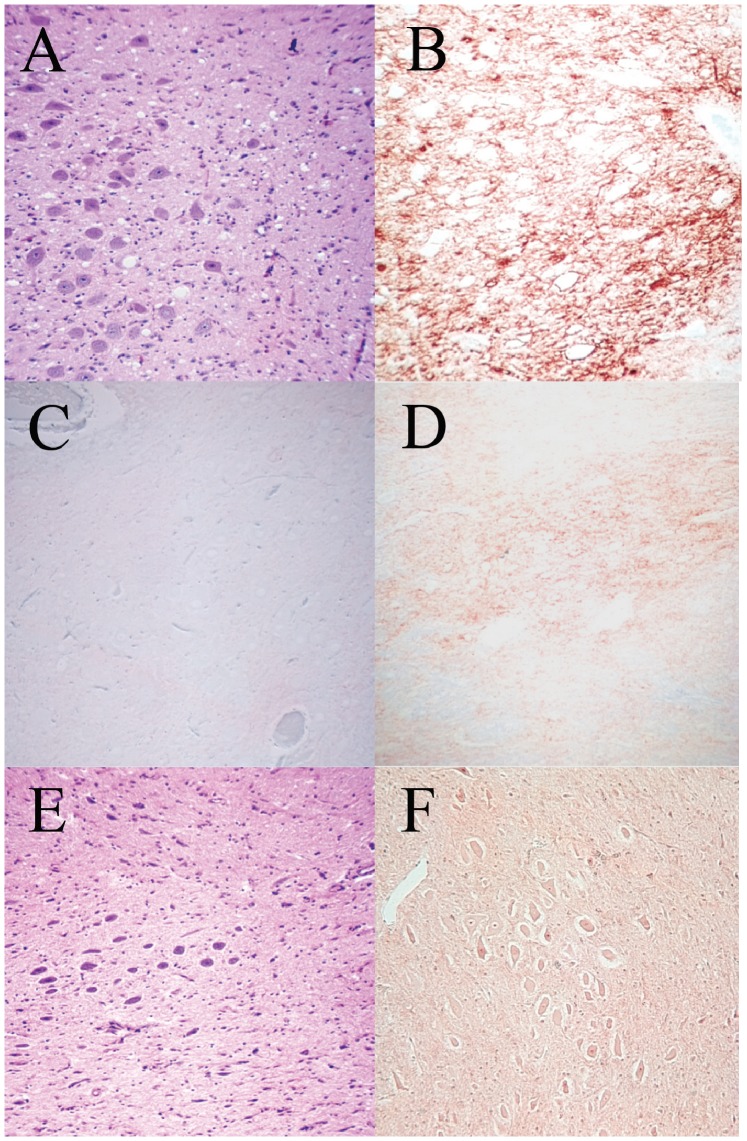
Central Nervous system pathology. Examples of vacuolation and immunostaining for PrP^Sc^ in brain tissue. A. Haematoxylin and Eosin (H&E) staining showing vacuolation in the dorsal motor vagus nucleus of L55 (VRQ/VRQ) had natural scrapie-like disease (x100). B. Strong immunostaining for PrP^Sc^ (diagnostic for natural scrapie) with BG4 antibody in the dorsal motor vagus nucleus of L55 (x40). C. Marginal immunostaining for PrP^Sc^ (diagnostic for SSBP/1) with BG4 in the dorsal motor vagus in the brain of L111 (VRQ/ARR) (x 100). D. Marginal immunostaining for PrP^Sc^ (diagnostic for SSBP/1) with BG4 in the dorsal motor vagus of L101 (VRQ/VRQ) (x100). E. Nil vacuolation (typical for SSBP/1) in an H&E section of the dorsal motor vagus in the brain of L101 (VRQ/VRQ) (x100). F. Marginal immunostaining for PrP^Sc^ (diagnostic for SSBP/1) in the brain of L106 (VRQ/VRQ) (x100).

Nineteen progeny resulted from Group 1 embryos, 12 born naturally and seven by caesarean delivery and hand rearing (CD). At least one copy of the VRQ allele was encoded by 14 progeny which were therefore susceptible to SSBP/1 and nine of this 14 were also of *PRNP* genotypes susceptible to NPU-natural scrapie. However only three of the latter group actually developed scrapie, two VRQ/VRQ (L55 and L64 at 846 and 954 days of age) and one VRQ/ARQ (L61 at 1863 days of age). All three had clinical signs, pathology and *PRNP* genotype indicative of natural scrapie which was confirmed by the presence of vacuolation (Figure 1A) and PrP^Sc^ detection by IHC which showed prominent staining of PrP^Sc^ in the brain stem, especially in the DMNV (Figure 1B) and peri-vascular PrP^Sc^ immunostaining in thalamic nuclei, both typical for natural scrapie [32]. Of this group of three animals, two originated from embryo donors that developed natural scrapie and one originated from a donor that died for other reasons. One case was born naturally (L64, VRQ/VRQ) but two cases (one each of VRQ/VRQ and VRQ/ARQ) were born by caesarean derivation (CD) and were hand-reared. Age at death from scrapie was much shorter in the VRQ/VRQ animals (846, 954 days) than in the VRQ/ARQ case (1863 days). Of the remaining progeny, 16 died (not scrapie) at ages between 1474 and 4907 days of age. Of these survivors, five with genotypes susceptible to natural scrapie (one VRQ/VRQ and four VRQ/ARQ) remained healthy with no sign of the disease to 2069–4268 days of age. None of the Group 1 progeny of this group showed any indication of SSBP/1 in clinical signs, pathology or *PRNP* genetics.

### Group 2: Scrapie Resistant SSBP/1 Challenged Recipient Group

Group 2 embryos were transferred into recipients challenged with SSBP/1 and of scrapie resistant genotypes. This group was designed to demonstrate whether or not SSBP/1 scrapie could transfer from sheep of resistant genotype to the offspring. Group 2 recipients were 16 animals of various AXX/AXX genotypes (Table 2). These recipients were not all fully genotyped as it was not a routine practice in the flock at that time (1994) and DNA is not available now to confirm. The sheep survived to an average age of 3216 days (SD 769 days, range 1493–4601 days) and, as expected because of their *PRNP* genotypes, none showed any signs either of natural scrapie or of experimental scrapie despite all being challenged with SSBP/1 at 26 days prior to the ET procedure being carried out and around 140–150 days prior to the birth of the transferred lambs. Survival average after SSBP/1 challenge was 1825 days (SD 704 days, range 1095–3285).

**Table 2:** Group 2: resistant recipients challenged with SSBP/1 scrapie and development of scrapie in offspring.

Donor	Recipient	Genotype	Death Other	Lamb born	Lamb	Genotype	Death scrapie	Death other
D2 (scr)*	R29	ARQ/ARQ	3125d	CD	L73	VRQ/VRQ**	834d	
D2 (scr)				CD	L74	ARQ/ARQ**		2443d
D2 (scr)	R30	ARQ/ARR	3960d	CD	L75	ARQ/ARQ		3706d
D2 (scr)	R31	ARQ/ARR	4016d	CD	L76	ARQ/ARQ		2915d
D2 (scr)				CD	L77	VRQ/ARQ	1975d	
D6	R32	ARQ/ARR	4601d	CD	L78	VRQ/ARQ		2684d
D6				CD	L79	VRQ/ARQ	1532d	
D7	R33	ARQ/ARR	2776d	NB	L80	VRQ/VRQ		4721d
D14	R34	ARQ/ARR	1493d	CD	L81	ARQ/AHQ		2490d
D8	R35	ARQ/ARR	3234d	NB	L82	VRQ/ARQ		4787d
D8				NB	L83	VRQ/ARQ		3760d
D7	R36	ARQ/AHQ	3210d	NB	L84	VRQ/VRQ		4718d
D7				NB	L85	VRQ/VRQ	1107d	
D14	R37	ARQ/AHQ	2033d	NB	L86	VRQ/AHQ		4805d
D2 (scr)	R38	AHQ/ARR	3256d	CD	L87	VRQ/VRQ	1018d	
D2 (scr)				CD	L88	VRQ/ARQ		3706d
D8	R39	AHQ/ARR	2860d	CD	L89	VRQ/VRQ	820d	
D8				CD	L90	VRQ/AHQ		2494d
D7	R40	ARR/ARR	3128d	NB	L91	VRQ/ARQ		3041d
D7				NB	L92	AHQ/ARQ		4012d
D14	R41	ARR/ARR	3191d	CD	L93	VRQ/AHQ		968d
D14				CD	L94	VRQ/ARQ		362d
D2 (scr)	R42	AXX/AXX	4132d	CD	L95	ARQ/ARQ		3789d
D2 (scr)				CD	L96	VRQ/ARQ		2441d
D2 (scr)	R43	AXX/AXX	3026d	CD	L97	ARQ/ARQ		3706d
D2 (scr)				CD	L98	VRQ/ARQ	2290d	
D6	R44	AXX/AXX	3407d	CD	L99	VRQ/ARQ		3706d
Totals								
5	16			8NB, 19CD		27	7	20

CD = caesarean derivation and hand rearing, NB = natural birth, reared by recipient ewe.

* (scr) – embryo donor later developed natural scrapie.

** Where two progeny indicated per recipient, these developed *in utero* together and born as “twins”. Deaths are from scrapie (Death scrapie) or other causes (Death other) and given in days of age (d).

Group 2 embryos transferred into the SSBP/1 challenged resistant recipient sheep resulted in 27 live lambs, 19 of which were born by CD, and eight born naturally. Seven lambs went on to develop signs of scrapie of which four originated from embryo donors that themselves developed natural scrapie and three originated from embryo donors that died for other reasons. Of the seven progeny which showed signs of scrapie, all were of genotypes (VRQ/VRQ or VRQ/ARQ) and pathology which suggested natural scrapie. Six animals had been born by CD (L73, L77, L79, L87, L89, L98) and one (L85) by natural birth. Survival times for CD animals were 820–1018 days for VRQ/VRQ (n = 3) and 1532–2290 for VRQ/ARQ (n = 3). The age at death from scrapie of the normal birth animal (VRQ/VRQ) was 1107 days, longer than the CD animals of the same genotype but within the normal range of such deaths in the NPU flock for this genotype [47]. In general VRQ/VRQ animals had shorter survival times (820–1107 days) than the VRQ/ARQ animals (1532–1975).

Of the 20 lambs which did not develop scrapie, one died at 362 days of age and the other 15 survived to ages ranging from 1975 to 4012 days. Of these survivors, 13 had genotypes encoding at least one VRQ allele including 10 with VRQ/VRQ or VRQ/ARQ which would predispose them to natural scrapie although this did not develop in the animals. The other seven offspring were all AXQ/AXQ. One of the scrapie susceptible VRQ/ARQ offspring (L88) died at 3706 days of age (not scrapie) despite developing *in utero* (R38, CD birth) with a VRQ/VRQ sibling (L87, same biological parents) which went on to develop natural scrapie. L88 had low level PrP^Sc^ staining in brain and tonsil but not the extensive staining associated with clinical scrapie. A further animal of VRQ/VRQ genotype (L84) survived to 4718 days of age despite developing *in utero* (R36, natural birth) with a sibling VRQ/VRQ lamb (L85) which went on to develop natural scrapie. Both L88 and L84 had low level staining for PrP^Sc^ in brain and L88 also showed some staining in tonsil. This may indicate sub clinical infection.

### Group 3: Scrapie Susceptible SSBP/1 Challenged Recipients

**Figure 2:**
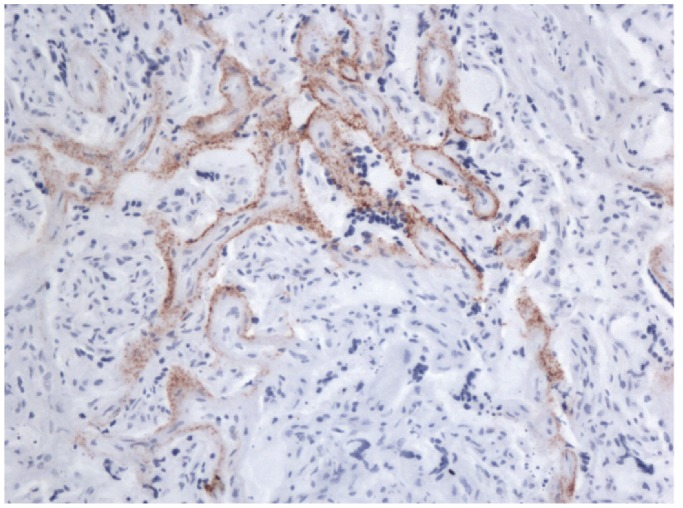
Strain typing lesion profiles of progeny scrapie cases in mice. Patterns of severity of vacuolation in nine grey matter areas (medulla, cerebellum, superior colliculus, hypothalamus, thalamus, hippocampus, septum, retrosplenial cortex and cingulate & motor cortex) and three white matter areas (cerebellum, superior cerebral peduncle, basal cerebral peduncle) in brain of tg338 mice challenged with brain from scrapie affected progeny. A: four progeny with lesion profiles similar to SSSBP/1 control. (B) One progeny with lesion profile similar to natural scrapie control.

Group 3 embryos (Table 3) were transferred into eight susceptible recipients, six of genotypes targeted by both natural scrapie and SSBP/1 (five VRQ/VRQ, one VRQ/ARQ) and two of genotypes targeted only by SSBP/1 (VRQ/AHQ and VRQ/ARR). In order to maximize the potential infectious dose to the embryos and resulting lambs, it was planned that challenge with SSBP/1 would produce scrapie clinical signs just after the birth of the lambs. Reflecting the longer incubation period in heterozygotes, VRQ/VRQ recipients were therefore injected about 150 days before they were due to lamb, and 13 days before embryos were transferred to them. The other recipients (VRQ heterozygotes) were injected about 270 days before being due to lamb and 111 days prior to embryo transfer. The results showed no clear association between time of birth and the time of development of clinical signs in the mother and so are not shown here. All eight recipients developed scrapie. The two sheep of genotypes susceptible to SSBP/1 but not natural scrapie (R51 and R52) each showed clear signs of SSBP/1-like clinical signs and pathology. The other six recipients of genotypes which are susceptible to both SSBP/1 and natural scrapie, could have had scrapie produced by either one of the strains or a combination of both. Three recipients (R45, R47 and R49) had clinical and pathology signs of natural scrapie whereas R46 had signs of SSBP/1. R50 and R48 had clinical phases of more than 40 days, on the long side for SSBP/1 but on the short side for natural scrapie and with clinical signs which were ambiguous. Pathology in these two animals were minor vacuolation, reminiscent of SSBP/1 but with some strong PrP^Sc^ staining as found with natural scrapie perhaps indicative of a mixed infection. Placental tissue from R46 stained positive for PrP^Sc^ (Figure 2) but very little testing of placental tissue in other animals was carried out.

**Table 3:** Susceptible recipients challenged with SSBP/1 and development of scrapie in offspring.

Donor	Recipient	Genotype	Age died	After SSBP/1*	Lamb born	Lamb	Genotype	Death scrapie	Death other	tg338 IP#
D3	R45	VRQ/VRQ	758d	150d	CD	L100	VRQ/ARR		4498 d	ND
D13	R46	VRQ/VRQ	841d	204d	CD	L101	VRQ/VRQ***	559 d		50dy (0.3)
D13					CD	L102	VRQ/ARR***	640 d		54dy (1.0)
D10	R47	VRQ/VRQ	749d	150d	CD	L103	VRQ/ARR		3384d	ND
D10					CD	L104	VRQ/AHQ		3977d	ND
D10	R48	VRQ/VRQ	778d	185d	CD	L105	VRQ/AHQ	1678d		ND
D5 (scr)**	R49	VRQ/VRQ	845d	201d	NB	L106	VRQ/VRQ	205d		53dy (0.5)
D10	R50	VRQ/ARQ	1518d	291d	CD	L107	AHQ/ARR		4498d	ND
D10					CD	L108	AHQ/ARR		4491d	ND
D10	R51	VRQ/AHQ	1248d	375d	CD	L109	VRQ/VRQ	807d		433dy (55)
D10					CD	L110	VRQ/ARR		3208d	ND
D13	R52	VRQ/ARR	1599d	368d	NB	L111	VRQ/ARR	429d		64dy (6.0)
D13					NB	L112	ARR/ARR		3506d	ND
Totals										
4	8				4NB, 9CD	13		6	7	

CD = caesarean derivation and hand rearing, NB = natural birth, reared by recipient ewe.

* After SSBP/1 = number of days till death following challenge with SSBP/1.

** (scr) – embryo donor later developed natural scrapie.

*** Where two progeny indicated per recipient, these developed *in utero* together and born as “twins”. Deaths are from scrapie (Death scrapie) or other causes (Death other) and given in days of age (d).

# Incubation period in days (dy) when strain typed in tg 338 mice (SD).

Group 3 embryos transferred into susceptible SSBP/1 challenged recipient mothers developed into 13 progeny. Six progeny developed scrapie of which three were born naturally (L105, L106 and L111) and three by CD with hand rearing (L101, L102 and L109). In general, the ages at death from scrapie were again younger in VRQ homozygotes than in heterozygotes but tended to be shorter than in Groups 1 and 2. With VRQ/VRQ animals ages at death were 205, 559 and 807 days and for the heterozygotes 429, 640 and 1678 days.

There was evidence of both natural scrapie and SSBP/1 in the affected progeny. One animal had clear signs of natural scrapie: (L109) of VRQ/VRQ genotype and survival time of 807 days, with classical signs of pruritus and associated wool loss and with natural scrapie-like pathology and PrP^Sc^ immunostaining in the brain. Of the other five scrapie affected offspring, three had genotypes which are not affected by natural scrapie in this flock: L102 and L111 (both VRQ/ARR) and L105 (VRQ/AHQ) and all of these three sheep had clinical signs, pathology and ICC staining for PrP^Sc^ typical of SSBP/1 infection. L111 was born naturally and had a short survival time, dying of scrapie at 429 days of age. Again like SSBP/1, L111 had no PrP^Sc^ immunostaining in the medulla (Figure 1C). The remaining two scrapie affected offspring (L101 and L106) had VRQ/VRQ genotypes but disease typical of SSBP/1. L101 (born by CD) and L106 (born naturally) both had very short clinical phase and SSBP/1-like pathology. L101 (Figure 1D and 1E) died at the early age of 559 days showing extremely mild PrP^Sc^ staining in medullary nuclei, including the dorsal vagus (Figure 1D). L106 was even younger when it developed scrapie and died aged 205 days showing no PrP^Sc^ in the medulla, very like SSBP/1 (Figure 1F). This is the shortest survival time for a non-experimentally infected scrapie-affected sheep recorded in more than 40 years study of the NPU flock.

The progeny which did not develop signs of scrapie (n = 7) survived to 3208–4498 days of age. Of the seven offspring which did not develop scrapie signs, four had genotypes encoding one VRQ allele, none of natural scrapie genotypes, and the other three included two AHQ/ARR sheep and one ARR/ARR. Four of these animals had no detectable IHC staining for PrP^Sc^, two sheep (L103 and L109, both VRQ/ARR) had diffuse positive staining in brain but not in peripheral tissues. These two sheep died without signs of clinical scrapie but it is possible they were subclinically infected with SSBP/1 as sheep of this genotype do not develop natural scrapie.

In summary therefore, out of 10 offspring with genotypes susceptible to SSBP/1, six developed scrapie and five of these showed clinical and pathological signs of SSBP/1. There were SSBP/1-like cases in the offspring born both naturally (n = 3) and by CD (n = 2) and the data for Group 3 is summarised and compared with the other groups in Tables 4 and 5.

**Table 4:** Summary of results, numbers of scrapie cases in each genotype group at risk from scrapie.

Scrapie strain infection risk	Genotypes of offspring	Group 1 (scr/n*)	Group 2 (scr/n)	Group 3 (scr/n)
At risk from both natural scrapie and SSBP/1	VRQ/VRQ & VRQ/ARQ	3/9	7/17	3/3
At risk from SSBP/1 only	VRQ/AHQ & VRQ/ARR	0/5	0/3	3/7

* scr/n = number of scrapie cases/total number at risk of infection.

**Table 5:** Summary of results: numbers of scrapie cases in each genotype group following caesarean derivation or normal birth.

		Group 1		Group 2		Group 3	
Scrapie strain infection risk	Genotypes of offspring	CD (scr/n*)	NB (scr/n)	CD (scr/n)	NB (scr/n)	CD (scr/n)	NB (scr/n)
At risk from both natural scrapie and SSBP/1	VRQ/VRQ & VRQ/ARQ	2/5	1/4	6/8	1/3	2/2	1/1
At risk from SSBP/1 only	VRQ/AHQ & VRQ/ARR	0/5	NA	0/2	0/1	2/6	1/1

* scr/n = number of scrapie cases/total number at risk of infection. CD = birth by caesarean section. NB = normal birth.

### Strain Typing Transmission to tg338 Mice

**Figure 3:**
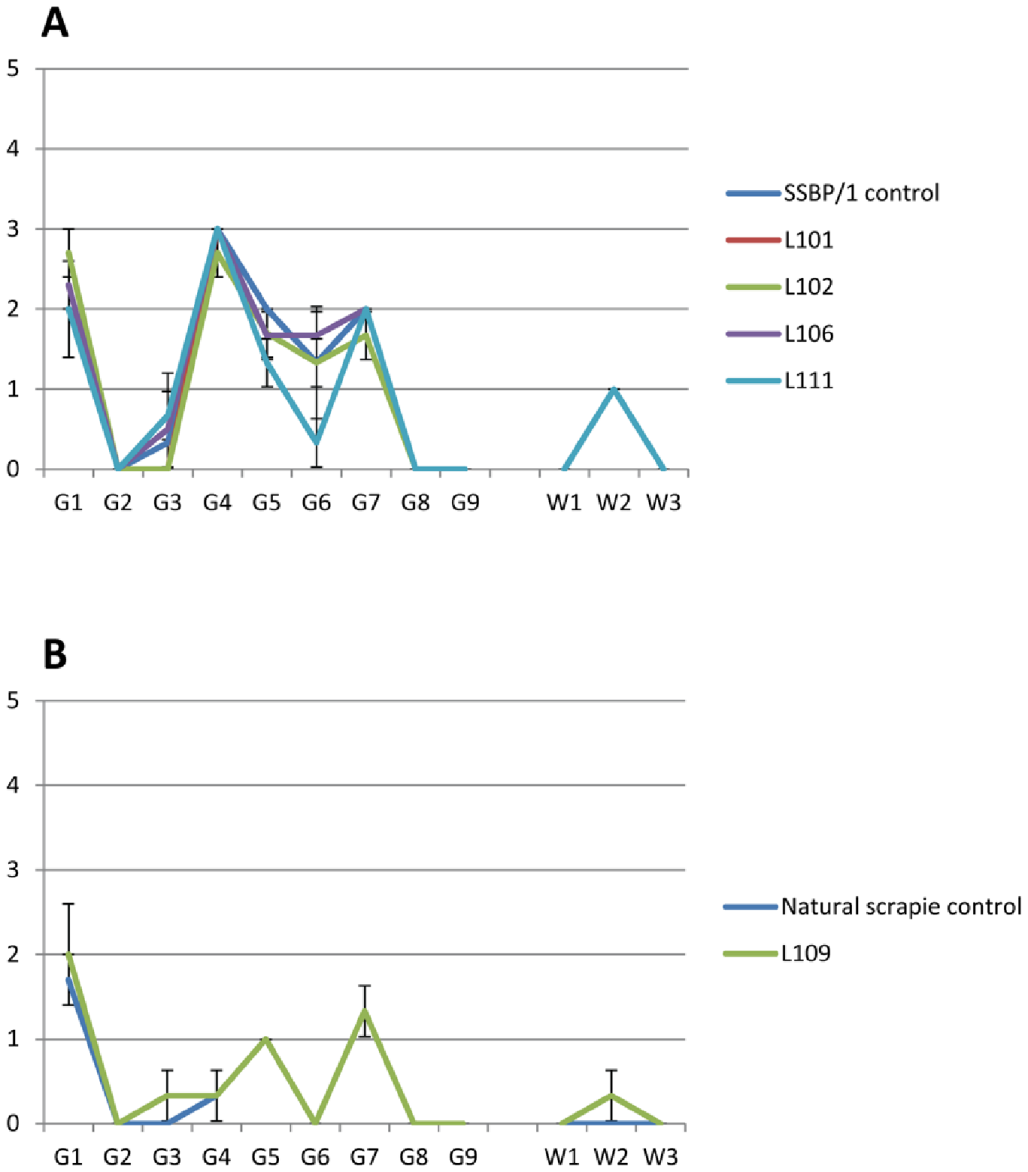
Placental tissue and PrP^Sc^. Immunostaining for PrP^Sc^ using BG4 antibody in placental tissue (cotyledon) from recipient ewe R46 (x 40).

Brain homogenates from the sheep L101, L102, L106, L109 and L111 were used for intracerebral inoculation of six each of tg338 mice, which carry the ovine VRQ allele [48]. The SSBP/1 control had an incubation period of 64 d (SD 3) in these mice, while the natural scrapie control from the NPU flock had an incubation period of 476 d (SD 18). L101, L102, L106 and L111 transmissions produced mean incubations of 50 d (SD 0.6), 54 d (SD 2.3), 53 d (SD1.3) and 64 d (SD 13.7) respectively with positive TSE histology. The sample from L109 produced an incubation period of 435 d (SD55). Lesion profiles for L101, L102, L106 and L111 were very similar to the SSBP/1 control, and L109 lesion profile was quite different and nearly identical to the NPU flock natural scrapie control (Figure 3).

### Western Blotting for PrP^Sc^ Detection

**Figure 4:**
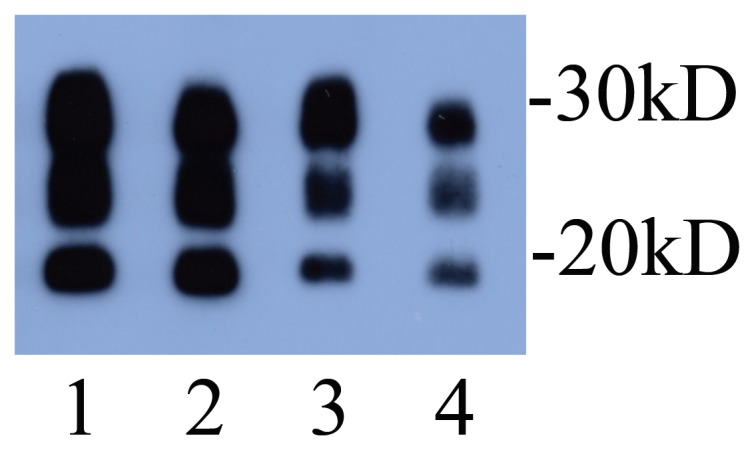
Western blot confirmation of scrapie infection. Examples of proteinase K treated samples from natural scrapie (lanes 1 & 3) and SSBP/1 (lanes 2 & 4). Loading: Lanes 1 and 2, equivalent to 20 mg starting brain wet weight; Lanes 3 and 4, equivalent to 6.67 mg starting brain wet weight.

Examples of the results of Western blotting for PrP^Sc^ in some of the scrapie-affected progeny and recipient ewes are shown in Figure 4. There is no reproducible difference between NPU natural scrapie and SSBP/1 however Western blotting confirms the diagnosis of scrapie. Figure 4 shows samples from sheep judged to have natural scrapie (lanes 1 and 3) and two from sheep judged to have SSBP/1 (lanes 2 and 4).

## Discussion

This study has examined the possibility of scrapie transmitting pre-natally between a pregnant, infected ewe and her lamb. The use of embryo transfer enabled embryos to be taken from sheep of specific *PRNP* genotypes and then transferred to surrogate mothers, which also had a range of genotypes thus extending the range of combinations of genotype and therefore susceptibility. Two groups of recipients were challenged with SSBP/1 scrapie, one group of resistant genotypes, the other of susceptible genotypes. None of the lambs were experimentally infected with scrapie, so any appearance of scrapie in these animals requires explanation.

In the offspring from Group 1 sheep (recipients not challenged with SSBP/1), natural scrapie occurred in 3 out of 19 offspring (16%) but with no signs of SSBP/1 scrapie in the offspring born either naturally or by CD from uninfected sheep of various PrP genotypes. The three scrapie cases which occurred in offspring resembled natural scrapie and two animals had biological mothers who themselves developed natural scrapie.

In the offspring from Group 2 sheep, (recipients of resistant genotypes but challenged with SSBP/1) scrapie occurred in 7 out of 27 offspring (26%), all in genotypes susceptible to natural scrapie. All these scrapie cases resembled natural scrapie and there was no indication specifically of SSBP/1 having transmitted to the lambs. Of the seven Group 2 offspring which died of scrapie, four (L73, L77, L87 and L98) were born by CD and were from a biological mother which herself developed natural scrapie (D2) but the other three had biological mothers which did not develop natural scrapie (D6, D7 and D8), one lamb born naturally (L85), two by CD (L79 and L89) suggesting infection in can occur after embryos were transferred and also before birth.

In the offspring of Group 3 sheep, (recipients of susceptible genotypes and challenged with SSBP/1) signs of SSBP/1 were seen in five out of the six offspring which died of scrapie, three born naturally (L105, L106 and L111) and two by CD (L101 and L102). Inoculation of brain from four of these (L101, L102, L106 and L111) into tg338 mice had short incubation periods and lesion profiles indistinguishable from SSBP/1. One of the offspring had clinical and pathology signs of natural scrapie (L109) and tissue from this animal generated incubation periods and a lesion profile in tg338 mice of 435 days similar to natural scrapie from the NPU flock. Within the offspring strain typed as having SSBP/1, two were very young when they died. L111 was 429 days of age but the other (L106) at 205 days of age is the youngest scrapie case ever recorded in 40 years of study in this flock in any animal outside of experimental challenges [2].

Our results are undoubtedly affected and rendered more difficult to interpret by the endemic natural scrapie in the flock, the incidence of which was not understood when the study was initiated. It is also true that caesarean derivation and complete separation from the ewe prior to birth did not prevent either classical or experimental scrapie appearing in the offspring. It is important when analysing the results to be absolutely clear about two factors, the first of which is the distinction between experimental (SSBP/1) and natural scrapie. There are a number of biological criteria which enable us clearly to distinguish the two forms of scrapie and these are presented in detail in the Methods section (Distinction between SSBP/1 and natural scrapie). In summary, natural scrapie disease signs and pathology are found only in NPU Cheviots of VRQ/VRQ and VRQ/ARQ genotypes [47]. SSBP/1 experimental scrapie affects the same genotypes but will also cause disease in VRQ/ARR and VRQ/AHQ genotypes in which natural scrapie has never been detected in this flock [38], [47]. It has been noted for over sixty years that SSBP/1 produces extremely low levels of vacuolation compared with natural scrapie [28], [29], [30], [31], [43], [57] and has different patterns of PrP^Sc^ distribution [44], [45] so we are confident of the identity of the strain of scrapie in each animal. However as always for TSE strain typing, bioassay in mice is the gold standard In this case five sheep brain homogenates were transmitted into tg338 mice for strain typing and confirmed the strain diagnosis by comparison with SSBP/1 and natural scrapie controls. SSBP/1 was shown to be a tg 338 rapid strain, very similar to those reported previously by other labs [50], [51], [52] and Figure 3 shows that it gives the expected lesion profile for these strains [49], the same as that produced from transmissions to tg 338 mice from four of our offspring sheep. One offspring produced a tg 338 lesion profile indistinguishable from the natural scrapie control and very different from SSBP/1. The second important factor to consider is that of the disease hygiene measures which were taken when caring for all of the sheep involved in this study, presented in Methods section (Lambing and sheep care). These were designed to prevent inadvertent contamination of the sheep with scrapie from other experiments running nearby and the success of these measures in preventing natural scrapie infection can be seen in the survival of three highly susceptible VRQ/VRQ offspring to 2069 days (L57, Group 1, Table 1), 4718 days (L84, Group 2, Table 2) and 4721 days (L80, Group 2, Table 2) when VRQ/VRQ animals in the breeding flock were developing natural scrapie at 800–900 days of age with 100% incidence [47]. There was also a considerably greater survival rate of VRQ/ARQ offspring in the experimental groups than out in the general flock. Where therefore did the SSBP/1 scrapie in the offspring originate? It could theoretically have infected the lambs as older animals by environmental contamination but in the history of the NPU Cheviot flock (>50 years), no other instances of transfer of SSBP/1 infection between animals, or sporadically occurring in animals, have ever been recorded (Hunter, unpublished). More specifically in the same 1994/1995 birth cohorts as the sheep in this study but in the main breeding flock, eight VRQ/AHQ sheep lived to 4–7 years (median 5 years) and 17 VRQ/ARR sheep lived to 4–12 years (median 6 years) with no sign of scrapie and although numbers are small, no other SSBP/1-like case was seen in lambs born in Groups 1 and 2 of this study.

It is difficult for us to see where the scrapie infection found in offspring could be coming from, except via the infected mothers. Caesarean derivation and complete separation of the lamb from its mother, no suckling exposure to maternal colostrum/milk but instead hand rearing by humans using New Zealand milk powder did not prevent either natural scrapie or SSBP/1 scrapie from appearing later in some of the lambs. Perhaps the process of removal of lambs from the ewes by laparotomy may have allowed them to be exposed to maternal blood, known to carry infection [58], however in skilled hands this process involves very little blood and lambs are removed very quickly from maternal contact so it is a low risk procedure. In our view these factors, taken together, strongly suggest that pre-natal, *in utero* transmission of infection may be occurring in these sheep and with two different strains of scrapie. The recent report of transmission of CWD between mother and offspring in Muntjac Deer lends support to this assertion [27]. Despite the species difference, there are similarities in pathogenesis between sheep and deer, for example the deposition of PrP^Sc^ in peripheral lymphoid tissues, which suggest there could be parallels also in mechanisms of transmission of infection.

If *in utero* transfer of infection can occur, by what means could it happen? Scrapie infectivity has been demonstrated in placental tissues [12], [13] and in this study we found mild PrP^Sc^ immunostaining in placenta from R46, with progeny L101 and L102 genotyped as VRQ/VRQ and VRQ/ARR, both of which developed scrapie with signs of SSBP/1 infection (Figure 2). Our results however are not surprising in the light of other reports which have shown that placental tissue in ewes incubating scrapie accumulates large amounts of PrP^Sc^ if the foetus has a susceptible *PRNP* genotype [14], [15] or is twinned with a susceptible sibling [16]. The placental structure in the pregnant sheep [59] makes it difficult to understand how infection might cross from ewe to lamb *in utero.* It is thought that the maternal/foetal barrier normally remains largely intact until birth although some blood borne infectious agents can traverse the placental barrier [60]. Diffusion across the maternal/foetus microvillus junction involves gaseous exchange together with other substances such as glucose and amino acids, which fulfil the foetal requirement for nutrition. However the studies which demonstrate PrP^Sc^ in sheep placental tissue [14], [15], [16] imply there must be some communication between mother and foetus for the reaction of PrP^Sc^ accumulation to take place. Interesting targets for further study would be hematomata, [61] which are regions within the placentomes, structures in which maternal and foetal membrane villi are juxtaposed. Hematomata, also known as haemophagous zones, are characteristic features of most carnivorous animals, are uncommon in ungulates but do occur as part of the cotyledons of small ruminants. These zones contain free maternal blood and are fully established in sheep by the 98^th^ day of gestation (full term ∼150 days). Photomicrographs of trophoblasts with internalized red blood cells [61], [62] suggest that these foetal-origin cells can phagocytose maternal blood cells. Others have suggested that haemophagous zones provide sources of iron for the foetus [63] and in theory could permit the transport of TSE infection between maternal blood and the unborn lamb. In other studies we have demonstrated conclusively that both scrapie and BSE infected sheep have high levels of infectivity in blood from early pre-clinical stages [58], [64], [65].

An alternate source of infection *in utero* is the amniotic fluid which has recently been shown to contain low levels of PrP^Sc^ in scrapie sheep [18]. Although it is less clear how amniotic fluid acquires PrP^Sc^, transfer of prions to the developing lamb could be achieved during foetal swallowing activity in the last third of gestation. Many mammalian species swallow *in utero*, and sheep foetuses in the last third of gestation have been shown to swallow 20–200 mls of fluid in 2–7 discrete episodes per day, every day [66]. It has been suggested that this activity may be helpful in regulating amniotic fluid contents or volume [67] and/or a simple development of swallowing ability which will be essential at birth. PrP^Sc^ has also been shown to be present in allantoic fluid [15]. This hypothesis fits well with the observation that PrP^Sc^ is first found in susceptible young lambs within Peyer’s patches and other alimentary tract tissues [14]. As often the case with TSE infection, there may be many different routes of infection operating together and our caesarean-derived lambs were clearly exposed to maternal blood in haemophagous zones, amniotic fluid during gestation and allantoic fluid during removal from the uterus – just as were the lambs born naturally.

In conclusion therefore, our study has presented evidence that suggests that transmission of scrapie can occur from the infected mother sheep to her lamb before birth, although it is almost certain not to be the only route by which a lamb can become infected. Further studies are clearly necessary in order to understand the underlying mechanisms and to be able to assess whether the very different placental structures in humans will protect babies from infection if born to CJD infected mothers. Other studies are near completion in our laboratory aimed at clarifying the rate of maternal transmission using scrapie-free sheep of New Zealand origin and therefore without the potential interference from natural scrapie. The ultimate aim of control and eradiation of scrapie infection in sheep clearly depends on understanding all the potential routes of transmission as the use of genetically resistant sheep is not always possible, especially for some rare breeds.

## Supporting Information

Table S1 **Biological parents of embryos and their fate.**
(DOCX)

Table S2 **Effect of washing of embryos on development of natural scrapie in resulting offspring.**
(DOCX)
