# Correction: Evidence in Sheep for Pre-Natal Transmission of Scrapie to Lambs from Infected Mothers

**DOI:** 10.1371/annotation/f7240569-aa33-4a9c-b7f0-75be4fad9999

**Published:** 2014-01-15

**Authors:** James D. Foster, Wilfred Goldmann, Nora Hunter

The legends of Figures 2 and 3 are switched. The order of the figures is correct.

