# Supplementary material for: Evidence in Sheep for Pre-Natal Transmission of Scrapie to Lambs from Infected Mothers
Source: PLoS One. 2013 Nov 18;8(11):e79433. doi: 10.1371/journal.pone.0079433 (PMC3832582; doi:10.1371/journal.pone.0079433)
Supplement: Table S1 — Biological parents of embryos and their fate. (DOCX) [file pone.0079433.s001.docx]

| **Donor ewe (n=15)** | **Genotype** | **Age at death (d)** | **Death after ET (d)** | **Pathology** | **Ram *(n=3)** |
| --- | --- | --- | --- | --- | --- |
| D1 (scr) | VRQ/VRQ | 837 | 174 | Natural Scrapie | A |
| D2 (scr) | VRQ/ARQ | 1216 | 218 | Natural Scrapie | A |
| D3 | VRQ/ARQ | 3595 | 2612 | Negative | B |
| D4 | VRQ/ARQ | 1605 | 955 | Negative | B |
| D5 (scr) | VRQ/ARQ | 1646 | 1321 | Natural Scrapie | B |
| D6 | VRQ/AHQ | 2350 | 1687 | Negative | A |
| D7 | VRQ/AHQ | 3977 | 3313 | Negative | A |
| D8 | VRQ/AHQ | 1635 | 1305 | Negative | A |
| D9 | VRQ/AHQ | 3001 | 2671 | Negative | B |
| D10 | VRQ/AHQ | 2931 | 1956 | Negative | B |
| D11 | VRQ/AHQ | 1181 | 202 | Negative | B |
| D12 | VRQ/AHQ | 1296 | 36 | Negative | C |
| D13 | VRQ/ARR | 2400 | 998 | Negative | B |
| D14 | ARQ/AHQ | 708 | 376 | Negative | A |
| D15 | ARQ/ARR | 3203 | 1961 | Negative | C |

Table S1. Biological parents of embryos and their fate.

* Rams did not develop natural scrapie. Ram genotypes (age at death, days) - A:VRQ/ARQ (2619), B:VRQ/ARR (2329),

C: VRQ/ARR (2360)
