# Supplementary material for: Evidence in Sheep for Pre-Natal Transmission of Scrapie to Lambs from Infected Mothers
Source: PLoS One. 2013 Nov 18;8(11):e79433. doi: 10.1371/journal.pone.0079433 (PMC3832582; doi:10.1371/journal.pone.0079433)
Supplement: Table S2 — Effect of washing of embryos on development of natural scrapie in resulting offspring. (DOCX) [file pone.0079433.s002.docx]

Table S2. Effect of washing of embryos on development of natural scrapie in resulting offspring.

| **IETS*** | **Embryos** | **Recipients** | **Scrapie donor, scrapie in offspring** | **Healthy donor, scrapie in offspring** |
| --- | --- | --- | --- | --- |
| Washed (n=17) | Resistant | Resistant*** | 0/4** | 0/4 |
|  |  | Susceptible | 0/0 | 0/0 |
|  |  |  |  |  |
|  | Susceptible | Resistant | 2/5 | 0/2 |
|  |  |  |  |  |
|  |  | Susceptible | 1/2 | 0/0 |
|  |  |  |  |  |
| Unwashed (n=42) | Resistant | Resistant | 0/10 | 1/8 |
|  |  |  |  |  |
|  |  | Susceptible | 0/0 | 0/5 |
|  |  |  |  |  |
|  | Susceptible | Resistant | 6/10 | 3/7 |
|  |  |  |  |  |
|  |  | Susceptible | 0/0 | 2/2 |
|  |  |  |  |  |

* International Embryo Transfer Society washing procedures

**scrapie cases/total offspring

*** Resistant and susceptible refer to natural scrapie associated *PRNP* genotypes, VRQ/VRQ and VRQ/ARQ
